# Pragmatic Integration of User-Centered Design and Implementation Science: A New Methodological Approach for Clinical Decision Support Implementation in EHRs

**DOI:** 10.1055/a-2716-4479

**Published:** 2025-10-30

**Authors:** Anna M. Maw, Jason A. Hoppe, Nicole M. Wagner, James Mitchell, Meagan Bean, Katy E. Trinkley

**Affiliations:** 1 Division of Hospital Medicine, University of Colorado Anschutz, Aurora, Colorado, United States; 2 Adult and Child Center for Outcomes Research and Delivery Science Center, University of Colorado Anschutz Medical Campus, Aurora, Colorado, United States; 3 Department of Emergency Medicine, University of Colorado School of Medicine, Aurora, Colorado, United States; 4 Department of General Internal Medicine, University of Colorado School of Medicine, Aurora, Colorado, United States; 5 Department of Biomedical Informatics, School of Medicine, University of Colorado Anschutz Medical Campus, Aurora, Colorado, United States; 6 Department of Family Medicine, School of Medicine, University of Colorado Anschutz Medical Campus, Aurora, Colorado, United States; 7 Colorado Center for Personalized Medicine, School of Medicine, University of Colorado Anschutz Medical Campus, Aurora, Colorado, United States

**Keywords:** user-centered design, clinical decision support, implementation science, case studies

## Abstract

**Background:**

Clinical decision support (CDS) tools are critical for improving care delivery and guideline adherence but are associated with clinician burnout when inadequately designed and implemented. User-centered design (UCD) and implementation science (IS) methods are evidence-based approaches to optimizing CDS tools, but are infrequently used in part due to limited guidance on how to apply them within resource-constrained health systems.

**Objective:**

This paper focuses on pragmatic application of an integrated UCD–IS approach, demonstrating how it can be adapted to meet operational constraints through two real-world case studies.

**Methods:**

We applied an integrated UCD–IS approach guided by the Practical Robust Implementation and Sustainability Model (PRISM) to two CDS projects within a large regional health system: (1) adapting a CDS for improving prescribing of goal-directed medical therapy in patients with heart failure during virtual visits, and (2) expanding a naloxone co-prescribing CDS across outpatient settings. Each project followed iterative phases—partner engagement, design, prototyping, deployment, and evaluation tailored to time and resource constraints of the health system. Methods used included interviews, focus groups, surveys, and usability testing.

**Results:**

Multilevel partner engagement surfaced critical insights that informed design adaptations. The heart failure CDS was adapted using minimal changes while the naloxone CDS underwent more extensive design iterations. Both projects balanced rigor and pragmatism, enabling timely implementation and rigorous design evaluation while supporting feasibility and sustainability. Iterative evaluations of both CDS are ongoing and structured to inform real-time refinements that support patient, clinician, and system-level outcomes.

**Conclusion:**

This work provides practical guidance on applying an integrated UCD–IS approach to CDS design and evaluation in time and resource–constrained health system environments. By flexibly applying this integrated approach, health systems can better address multilevel partner needs, ensure contextual relevance, and support sustained adoption.

## Background and Significance

Poorly designed clinical decision support (CDS) systems can lead to low adoption and alert fatigue, and have significantly contributed to widespread burnout in primary care physicians. 1 2 Two fields that can improve the adoption, user experience, and effectiveness of CDS are user-centered design (UCD) and implementation science (IS). To understand their unique contributions to addressing this challenge, it is important to understand the origins, scope, and emphasis of both methodological fields. UCD emerged in the 1980s and is rooted in human–computer interaction and cognitive psychology. It emphasizes optimizing usability, workflow integration, and user satisfaction through methods such as usability testing, task analysis, and participatory design. 3 4 In contrast, IS emerged in the early 2000s from public health and behavioral science to address system-level adoption, sustainability, and scalability of evidence-based interventions guided by frameworks like Practical Robust Implementation and Sustainability Model (PRISM) 5 and Consolidated Framework for Implementation Research (CFIR) 6 that account for multilevel contextual factors, including organizational readiness and policy alignment. 7 Although both disciplines value stakeholder engagement, iterative development, and have the capacity to work at both outer and inner levels of context, UCD most often operates at the micro-level (e.g., user interface, task flow), whereas IS tends to emphasize macro-level considerations such as organizational culture and external policy. This distinction, well-articulated by Dopp et al, 8 underscores that integrating UCD and IS offers a more consistently comprehensive and pragmatic approach to CDS development. This integrated approach aligns closely with the aspirational vision of Learning Health Systems, which seek continuous improvement in healthcare through real-time knowledge generation and the seamless integration of evidence and clinical practice. 9 By merging the user-driven focus of UCD with the rigorous and diverse partner-informed, context-sensitive implementation and evaluation methodologies of IS, CDS can more effectively address both the immediate usability needs of end users as well as dynamic multilevel implementation and robust evaluation considerations.

Although UCD and IS are evidence-based methods to improve CDS adoption and effectiveness of changing behavior, 4 5 6 7 they are not consistently applied, in part because of the time and resources required within already resource-constrained health systems. 8 9 Some health systems may not have the UCD or IS expertise, or may perceive UCD or IS methods to be rigid and not practical or feasible to apply given the many competing health system priorities. 10 11 12 13 Despite misconceptions, 14 the field of IS prioritizes “pragmatic” methods and making its methods more approachable and accessible to diverse audiences, including those with limited or no IS expertise and low resource settings. 5 15 Central to making these methods more accessible is providing guidance through examples of how they can be practically applied, including when used together as a unified approach with UCD—an *integrated UCD* – *IS* approach.

## Objectives

Although previous work has detailed the theoretical underpinnings and approaches for combining methodologies from these fields 8 16 and demonstrated their effectiveness, 4 5 6 7 we have yet to observe examples of practical applications that transparently exhibit how an integrated UCD–IS approach can flex to meet the challenges of operational resource constraints and timelines. 17 Therefore, the objective of this paper is to bridge this gap by providing two real-world examples that reveal practical insights in applying a UCD–IS integrated approach to design, and evaluate CDS across diverse contexts and resource constraints. The work described in the examples each occurred over the course of a year, with periods of inactivity at times.

## Methods

**Fig. 1:**
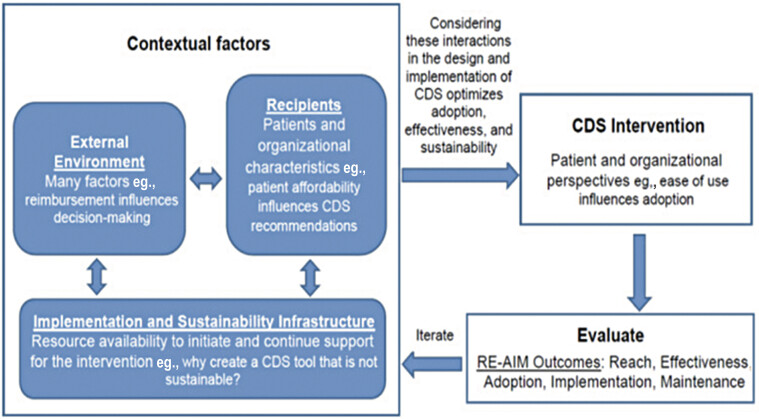
Domains of the Practical Robust Implementation and Sustainability Model (PRISM), their interactions, and how they influence clinical decision support (CDS). Adapted from Trinkley KE, Kahn MG, Bennett TD, et al. Integrating the Practical Robust Implementation and Sustainability Model with best practices in clinical decision support design: implementation science approach. J Med Internet Res. 2020;22(10):e19676.

**Fig. 2:**
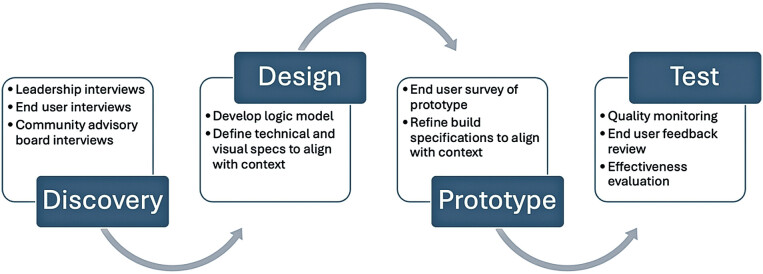
Iterative user-centered design clinical decision support (CDS) tool development process.

We applied an integrated UCD–IS approach to guide the design and evaluation of two CDS. 8 Use of a determinant and evaluation framework to inform the assessment and alignment of the CDS with the multilevel context is central to applying IS. As described in prior work, we used PRISM as the guiding framework for both CDS examples ( Fig. 1 ). 8 In Table 1 , we briefly summarize this process for applying the integrated UCD–IS approach, describing how the IS phases align with the iterative Discover, Design, Prototype, and Test steps ( Fig. 2 ) commonly used in the UCD literature. 18 In Table 1 , we also contrast the contributions of IS and UCD at each phase and highlight some common methods used at each phase.

**Table 1:** Implementation science phases and user-centered design steps of CDS development

Phase	Implementation science (IS) phase	User-centered design (UCD) step	Key differences	Typical methods
1	Multilevel partner engagement	Discover	IS: Emphasizes broad multilevel partner involvement (clinicians, admin, IT). Guided by determinant IS frameworks (e.g., contextual domains of PRISM). Often focuses on qualitative assessments. UCD: Primarily focuses on detailed user research to understand user tasks and workflows, often includes qualitative and quantitative assessments.	IS: Apply determinant framework and qualitative methods to understand multilevel contextual factors that affect implementation and effectiveness. 19 UCD: Quantitative methods: User surveys workflow analyses, user activity data collection. Qualitative methods: User interviews, and observations to understand user needs and challenges. 20 21
2	Designing the CDS	Design	IS: More focused on ensuring alignment with clinical workflows and organization. Prioritizes interventions that are equitable, sustainable, and adaptable for future scalability beyond the local setting. UCD: Applies human–computer interaction principles with end-user input. Develops multiple iterations based on design best practice with a focus on designing for the average/most common user.	IS: Diverse partner interviews guided by a IS determinant framework to understand multilevel contextual determinants. UCD: Brainstorming, parallel design, storyboarding, affinity diagram, card sorting, paper prototyping, software prototyping, etc. 20 21
3	Design and usability testing	Prototype	IS: Emphasizes evaluating feasibility and alignment with both end users and the broader clinical and system-level goals. UCD: Emphasizes feasibility and alignment with end users. Concentrates on usability, intuitive interfaces, and task optimization.	IS: Iterative prototyping and simulated testing to optimize multilevel partner priorities. 8 UCD: Iterative prototyping and simulated testing to optimize user experience. 18
4	Thoughtful deployment		IS: Focuses on controlled, pragmatic rollout with attention to unintended consequences and organizational readiness (training, support). UCD: Continuous prototype iterations but with increasing fidelity in real-world conditions and may include A/B testing designs.	IS: Logic model development and selection of implementation strategies tailored to address implementation barriers identified by diverse multilevel partners in phase 1. 25 26 UCD: Pragmatic study designs with plans to assess for adaptations needed to maximize user adoption. 20 21
5	Performance evaluation and maintenance	Test	IS: Guided by IS evaluation frameworks (e.g., RE-AIM domains of PRISM). Concentrates on evaluations of clinical effectiveness, implementation (i.e., measures of reach, adoption, fidelity, adaptation, cost, sustainment), and representativeness. UCD: Focuses on final usability enhancements, user satisfaction, and engagement.	IS: Iterative evaluation of dynamic context and implementation and effectiveness outcomes with implementation strategies adapted and deployed based on this interval data to enhance effectiveness, adoption, reach, sustainability, and scalability. 11 UCD: Quality monitoring to assess technical issues and end-user engagement to identify opportunities to improve use. 20 21

Abbreviations: CDS, clinical decision support; PRISM, Practical Robust Implementation and Sustainability Model; RE-AIM, reach, effectiveness, adoption, implementation, maintenance.

### Practical Application: Case Studies

To illustrate the practical application of integrating IS and UCD, we present two case studies that demonstrate how this approach can be used to design CDS in a manner that is adaptable and highly feasible in real-world health system environments. These examples highlight how this integrated approach was adapted to assess and align technology with the local context and the outcomes achieved. Importantly, they illustrate when and how deviations or adaptations from “traditional” IS or UCD methods are made in response to operational priorities and resource constraints. Both examples occurred in the same large regional health system spanning Colorado, Wyoming, and Nebraska, representing 14 hospitals and more than 150 clinics across suburban, urban, community, and academic settings. The health system uses one integrated electronic health record (EHR) instance (Epic systems) for all care settings.

#### Case Study 1: Heart Failure CDS Project

The objective of this project was to adapt a CDS tool to enhance the prescribing of guideline-directed medical therapy in patients with heart failure with reduced ejection fraction during virtual primary care and cardiology clinic visits across the entire health system. CDS was already developed for in-person visits but was not designed to address the unique workflows of virtual visits. The existing CDS was an interruptive alert designed using the integrated UCD–IS approach, which included extensive user-centered and multilevel partner engagement culminating in simulated usability testing. In this case, there was an urgency to design the CDS for virtual visits to capitalize on resources that would be available for evaluation.

#### Case Study 2: Naloxone CDS Project

The objective of this project was to expand existing CDS to improve 2022 CDC opioid prescribing guideline–recommended naloxone co-prescribing across all care settings in the health system. 19 The previously developed CDS, an interruptive alert available in the inpatient and emergency department settings, needed to be updated based on new guidelines and to address differing workflows and contextual factors in outpatient care. In this case, based on inpatient and emergency department data, operational leaders were asking for the new CDS to be implemented quickly to improve patient care.

## Results

### Phase 1: Multilevel Partner Engagement

#### Case Study 1 (Heart Failure with Reduced Ejection Fraction Telehealth Example)

In the heart failure example, multilevel partner engagement was informed by prior work and focused input on contextual information specific to virtual visits was also elicited. Partner engagement started with 30-minute semi-structured interviews of health system leaders representing cardiology, digital health, population health, and an executive leader for informatics operations. We sought input from the health system leader to understand organizational drivers of virtual visits. Additionally, we performed 30-minute semi-structured interviews with 10 primary care clinicians, 10 cardiology clinicians, and 10 patients. Through clinician interviews we aimed to understand barriers and facilitators to virtual visits for managing guideline-directed medical therapy. Through patient interviews we aimed to understand their perspectives on virtual visits, including barriers and facilitators to leveraging these visits. The interview guides were informed by PRISM to ensure consideration of the multilevel context. Via multilevel partner engagement we identified the need for clinicians to have access to current measurements of patient vitals and weights so that they could make informed decisions about guideline-directed medical therapy management. Further, patients reported needing guidance on how to share information (e.g., vitals, weights) asynchronously with their clinician when asked.

#### Case Study 2 (Naloxone Example)

The iterative process for tool development ( Fig. 2 ) began with interviews of 12 health system leaders representing department and quality leadership, executive leaders overseeing informatics and clinical operations and the program manager for CDS tool. Next, we conducted 5 focus groups with providers (25 providers total) from the inpatient and outpatient settings to determine context-specific facilitators and barriers to successful workflow integration, and to identify any additional implementation strategies to improve adoption. We then facilitated a discussion with a standing community advisory board (12 patients total) to obtain patient perspectives and ensure the CDS objectives supported patients' needs. Through multilevel engagement we identified a general need for additional education on guideline-concordant care and the need for the CDS to address unique outpatient practice patterns.

### Phase 2: Designing the CDS

#### Case Study 1 (Heart Failure with Reduced Ejection Fraction Telehealth Example)

Based on the multilevel partner input from Phase 1, we considered what was technically feasible and applied principles of human–computer interaction to adapt the design of the existing CDS. Due to technical feasibility and resource constraints in getting patients bluetooth-enabled devices, we were unable to automate the collection and sharing of patient home measurement data. Instead, we created provider and patient instructions on how to order and upload patient-reported home vitals and added these as hyperlinks within the CDS.

#### Case Study 2 (Naloxone Example)

Based on feedback from our multilevel partners, we refined the CDS content. Specifically, we: (1) revised the message language to incorporate more detailed information on why the CDS was triggered, and (2) created provider response options specific for acute care versus outpatient care settings.

### Phase 3: Design and Usability

#### Case Study 1 (Heart Failure with Reduced Ejection Fraction Telehealth Example)

We created three low fidelity prototypes of the revised CDS interface using PowerPoint software. We used these prototypes to conduct abbreviated design testing with cardiology leaders to ensure the content was acceptable. Based on input from cardiology leaders, one near-final prototype was created. This prototype was reviewed by the health system CDS governance committee who suggested using newly available technology to make the links expandable to save space, which was incorporated into the final prototype that then underwent usability testing. For usability testing, the research team created test patients in the EHR and checked to ensure the links worked, telehealth workflows remained smooth, and nothing was broken. Design and usability testing was limited in scope and did not include standard simulated testing with end users because adaptations to the existing heart failure CDS were minimal and did not alter the logic or overall clinician workflow. The abbreviated design and usability testing was also in response to the operational need to deploy the adapted CDS quickly.

#### Case Study 2 (Naloxone Example)

We developed two low fidelity prototypes of the CDS per setting (a total of four prototypes) using PowerPoint software. Design testing started with sharing the prototypes during a standing CDS governance meeting to obtain input on the response options and wording. Based on input from the CDS governance group, we developed a short survey for potential end users to respond to specific design questions related to provider response options, duration of suppressing the alert if dismissed, and how much detail to provide regarding the reason the CDS was recommending a naloxone prescription. This survey also included a validated question about acceptability and open-ended response options for general feedback. The survey was created using Qualtrics and distributed to 67 potential end users. A total of seven outpatient and eight acute care providers responded to the survey—a response rate of 22%. The survey led to setting specific changes. To improve adoption and workflow integration related to continuity of care in primary care, we added a bypass option to remind the clinician at the next visit. This option was suggested to appreciate the potential benefit of the recommendation while acknowledging the limited time and need for prioritization during clinic visits. The CDS language was also modified to clarify the reasons for recommending naloxone. Given end users' requests to include more detail about why the CDS was recommending naloxone, the research team considered listing patient-specific risk factors within the alert language instead of listing the general risk factors; however, given the additional technical resources required to create patient-specific messages, it was decided that a simple list of general risk factor conditions in the CDS messaging was pragmatic.

### Phase 4: Thoughtful Deployment (with Attention to Context)

#### Case Study 1 (Heart Failure with Reduced Ejection Fraction Telehealth Example)

The initial deployment plan for the revised CDS was to turn it on for all outpatient cardiology virtual visits. However, to generate new knowledge regarding best practices for virtual visits, we decided to randomize half of the outpatient cardiology clinics to the revised CDS and the other to the existing heart failure CDS to allow for a comparison. We selected outpatient cardiology because there was a previously expressed desire to have CDS for virtual visits and the leaders were supportive. The timing of the deployment was strategic to coincide with availability of resources to evaluate the impact of the change. Prior to deployment, a health system–wide newsletter sent to clinicians announced the deployment.

#### Case Study 2 (Naloxone Example)

Operational leaders requested full system roll out based on the success of the existing CDS that was successfully deployed in inpatient and emergency department settings 20 ; thus, traditional clinic or provider-level randomization was not considered feasible. The CDS was deployed in outpatient settings using a stepped-wedge cluster randomized trial across the health system. Based on our multilevel partner engagement, we identified feasible supporting implementation strategies to address potential barriers to the CDS. Notably, we determined the need to disseminate educational information separate from and in addition to the CDS. Thus, prior to CDS deployment we disseminated education via (1) standing clinic meetings and email announcements and (2) a health system–wide clinician education newsletter.

### Phase 5: Evaluation and Maintenance

#### Case 1 (Heart Failure with Reduced Ejection Fraction Telehealth Example)

The randomized controlled trial is currently in progress. At the end of 6 months, we will conduct a mixed methods assessment comparing the adapted CDS to the active control. The evaluation will be guided by both the contextual and RE-AIM (reach, effectiveness, adoption, implementation, maintenance) domains of PRISM to consider the influence of the context on both implementation and effectiveness outcomes. We will iteratively conduct semi-structured interviews of clinicians to understand the quantitative findings, including contextual drivers of implementation.

#### Case Study 2 (Naloxone Example)

Evaluation included outcomes across RE-AIM domains. During initial deployment quality monitoring and feedback, we are using reviews from clinicians to ensure technical function and refine the tool for improved adoption and effectiveness. At completion of 6 months, we will assess the effectiveness of the CDS and education on patient's receipt of guidelines-concordant care in the CDS versus usual care while accounting for secular trends.

The contents of Table 2 are based on our professional experience and describe methodological decisions that frequently arise when applying an integrated UCD–IS approach to CDS implementation as well as the advantages and potential tradeoffs, including implications for adoption, feasibility, equity, and sustainability.

**Table 2:** Methodological decisions and their implications for adoption, feasibility, equity, and sustainability

Methodologic decision	Advantages	Potential tradeoff	Implications for adoption	Implications for feasibility	Implications for equity	Implications for sustainability
Pragmatic multilevel partner engagement and co-development (early and iterative)	Builds trust and shared mental model of approach and goals; improves contextual fit	Requires time, effort, and coordination	Improves end-user experience	Requires investment in time establishing and maintaining a collaborative relationship	Engages diverse voices meaningfully	Builds strong foundation for long-term iterative adaptions that will be required for maintenance
Pragmatic “right-sized” UCD steps based on context	Increases feasibility in real-world health system environments	Risk of overlooking usability issues	Supports operational goals and responsiveness	Highly feasible; fits within system resources and can expedite timelines in fast-paced environments	May underrepresent needs of some users if explicit attention is not given to equity	Allows implementation under tight constraints
Pragmatic mixed methods evaluation guided by IS framework	Captures broad impact including equity, cost, and reach	Requires specialized expertise and coordination	Supports comprehensive understanding of the intervention's value	Resource-intensive but produces actionable insights	Framework explicitly accounts for equity and representativeness	Promotes structured learning and sustainability
Deployment of a minimum viable product with iterative refinement	Enables rapid deployment, and increases implementation speed	May launch with usability or equity gaps or unintended consequences if unchecked	Allows early clinician exposure and buy-in	Highly feasible, scalable, and aligns with learning health systems principles	Iteration allows for adjustment if disparities or other unintended consequences are detected	Encourages a culture of continuous improvement
Embedded deployment strategy matched to leadership support	Ensures alignment with strategic priorities	May limit generalizability or create implementation bias	Supports organizational readiness	More feasible than full randomized controlled trials; balances rigor with practicality	Targeted deployment can miss harder-to-reach populations	Structured rollouts support ongoing learning and refinement

Abbreviations: IS, implementation science; UCD, user-centered design.

## Discussion

Here we offer two case studies illustrating the pragmatic integration of UCD and IS methods in developing and evaluating CDS systems within a real-world healthcare setting. Both cases highlight the tension between creating an ideal, extensively tested CDS and delivering a minimum viable product 21 that meets immediate clinical or operational demands within existing resource constraints. Our examples reinforce a core principle shared by both UCD and IS: incremental, iterative development and evaluation is more feasible and preferable to prolonged pursuit of a “perfect” product that may rapidly become outdated due to evolving care models and patient needs.

While prior work has described theoretical alignments of UCD and IS, 8 16 22 23 the current work offers detailed illustrations of how these methodologies can be flexibly integrated. For instance, the naloxone example effectively utilized focus groups and iterative surveys within existing organizational structures, whereas the heart failure project employed individual interviews and streamlined usability testing, given limited timelines and minimal needed adjustments. Both examples demonstrate the value of continuous evaluation and adaptation informed by multilevel partner feedback, emphasizing that iterative evaluation is a powerful tool to maintain rigor, and achieve and sustain contextual fit in a rapidly evolving environment.

Consistently applying an integrated UCD–IS approach can present multiple challenges, including resource and expertise constraints. A future goal then is to continue to evolve these techniques to make them increasingly pragmatic, as well as disseminate knowledge and skill of these approaches to those responsible for deploying CDS within health systems. These techniques can then become accepted as both best practice *and* as feasible to perform within the constraints of usual operating procedures.

The integration of UCD and IS methods aligns closely with learning health system principles, which prioritize continuous, data-driven improvements embedded into routine practice. The iterative and adaptive approach described here mirrors the learning health systems' emphasis on systematically harnessing clinical data and feedback loops to enhance healthcare delivery and patient outcomes. Specifically, the iterative nature of UCD–IS parallels the concept of rapid-cycle learning inherent to learning health systems, exemplified in prior successful implementations. 24 Furthermore, by fostering multilevel partner engagement, this integrated approach operationalizes the learning health systems' tenet of participatory governance and partner-driven improvement, consistent with best practices. Thus, the pragmatic integration of UCD and IS not only addresses immediate clinical and operational demands but also contributes structurally and conceptually to the evolving healthcare landscape increasingly guided by learning health systems principles.

### Strengths and Limitations

A notable strength of this integrated UCD–IS approach is its flexibility. Our two case examples—one focusing on a rapid modification of an existing CDS for heart failure telehealth and another emphasizing more robust iterative testing for naloxone prescribing—demonstrate how the same overarching framework can be adapted to different clinical contexts, timelines, and resource constraints. This adaptability makes the approach widely generalizable and highly relevant to real-world operational challenges and environments. However, decisions regarding the extent of partner engagement or whether to skip or shorten UCD steps or formal qualitative methods to fit with health system timelines and available resources need to be thoughtful to avoid unanticipated consequences and ensure the appropriate scope of generalizability is preserved. Although this approach aims to be responsive to situations when IS or UCD expertise may be absent, organizations without such expertise may currently find it challenging to apply this integrated approach or determine when, how, and to what extent the approach can be modified. As such, this paper serves to provide concrete examples of different ways this approach can be successfully modified. We encourage dissemination of additional examples.

## Conclusion

The integration of UCD with IS offers a feasible approach to developing, implementing, and evaluating CDS that can improve the translation of evidence into practice. Adopting a flexible, iterative, evaluative mindset and leveraging the strengths of both fields can enhance the relevance, sustainability, and usability of these technologies in a maximally feasible manner. This new and pragmatic integration of disciplines provides a framework for understanding and applying these principles in real-world settings, ultimately improving the impact and success of CDS. This manuscript offers two pragmatic examples of how UCD and IS methods can be integrated to support CDS implementation in real-world health systems. Although this pragmatic approach has potential to improve uptake of these evidence-based methods to optimize CDS solutions, additional examples of practical application across diverse settings are needed. Future work should focus on continually improving the feasibility of applying this integrated approach to CDS implementation so that it can be used by health systems with a range of expertise, resource constraints, and operational priorities across diverse clinical scenarios.

## Clinical Relevance Statement

The pragmatic integration of IS and UCD in the design of CDS provides a practical framework for applying these principles in real-world context, thus improving the potential impact of CDS and facilitating the translation of evidence into practice.

## Multiple Choice Questions

1. Which one of the following is an advantage of using implementation science with traditional user-centered design methods?
  - Alignment with organizational priorities
  - Alignment with end-user priorities
  - Integration with end-user workflows
  - Integration of interface usability principles
  **Correct Answer** : The correct answer is option a is correct. Although UCD approaches are capable of considering broader contextual factors, UCD methods most often focus on the assessment of and alignment with end-user preferences, priorities, and workflows and includes integration of human–computer interaction principles to optimize interface usability. In contrast, implementation science consistently prioritizes contextual drivers beyond the end user, including organizational priorities.
2. Which one of the following is a principle shared by both implementation science and user-centered design?
  - Aim for a perfect solution
  - Aim for a minimum viable product
  - Avoid incremental, iterative development
  - Avoid solutions with potential to be quickly outdated
  **Correct Answer** : The correct answer is option b is correct. Both implementation science and user-centered design acknowledge that most interventions and the context in which they are deployed are complex and dynamically change over time because of advances in technology and shifts in healthcare. Given the speed of change, aiming for a “perfect” solution is not likely to be feasible and can delay progress. Aiming for a minimum viable product with plans to iterate is the goal.

## References

[1] Jankovic I Chen J H Clinical decision support and implications for the clinician burnout crisis Yearb Med Inform 2020 29 01 145 154 32823308 10.1055/s-0040-1701986 PMC7442505

[2] Budd J Burnout related to electronic health record use in primary care J Prim Care Community Health 2023 14 21501319231166921 10.1177/21501319231166921 PMC10134123 37073905

[3] Abras C Maloney-Krichmar D Preece J Encyclopedia of Human-Computer InteractionBainbrige, W: Thousand Oaks.Sage Publications 2004 445 456

[4] Maguire M Methods to support human-centred design Int J Hum Comput Stud 2001 55 04 587 634

[5] Glasgow R E Trinkley K E Ford B Rabin B A The application and evolution of the Practical, Robust Implementation and Sustainability Model (PRISM): history and innovations Glob Implement Res Appl 2024 4 04 404 420 39568619 10.1007/s43477-024-00134-6 PMC11573842

[6] Damschroder L J Reardon C M Widerquist M AO Lowery J The updated Consolidated Framework for Implementation Research based on user feedback Implement Sci 2022 17 01 75 36309746 10.1186/s13012-022-01245-0 PMC9617234

[7] Brownson R C Colditz G A Proctor E K Dissemination and Implementation Research in Health: Translating Science to Practice Oxford University Press 2023

[8] Dopp A R Parisi K E Munson S A Lyon A R Aligning implementation and user-centered design strategies to enhance the impact of health services: results from a concept mapping study Implement Sci Commun 2020 1 01 17 32885179 10.1186/s43058-020-00020-w PMC7427975

[9] Greene S M Reid R J Larson E B Implementing the learning health system: from concept to action Ann Intern Med 2012 157 03 207 210 22868839 10.7326/0003-4819-157-3-201208070-00012

[10] Shakowski C Page Ii R L Wright G Comparative effectiveness of generic commercial versus locally customized clinical decision support tools to reduce prescription of nonsteroidal anti-inflammatory drugs for patients with heart failure J Am Med Inform Assoc 2023 30 09 1516 1525 37352404 10.1093/jamia/ocad109 PMC10436140

[11] Trinkley K E Kroehl M E Kahn M G Applying clinical decision support design best practices with the practical robust implementation and sustainability model versus reliance on commercially available clinical decision support tools: randomized controlled trial JMIR Med Inform 2021 9 03 e24359 33749610 10.2196/24359 PMC8077777

[12] Brunner J Chuang E Goldzweig C Cain C L Sugar C Yano E M User-centered design to improve clinical decision support in primary care Int J Med Inform 2017 104 56 64 28599817 10.1016/j.ijmedinf.2017.05.004 PMC5836500

[13] Luna D R Rizzato Lede D A Otero C M Risk M R González Bernaldo de Quirós F User-centered design improves the usability of drug-drug interaction alerts: experimental comparison of interfaces J Biomed Inform 2017 66 204 213 28108211 10.1016/j.jbi.2017.01.009

[14] Beidas R S Dorsey S Lewis C C Promises and pitfalls in implementation science from the perspective of US-based researchers: learning from a pre-mortem Implement Sci 2022 17 01 55 35964095 10.1186/s13012-022-01226-3 PMC9375077

[15] Trinkley K E Glasgow R E D'Mello S Fort M P Ford B Rabin B A The iPRISM webtool: an interactive tool to pragmatically guide the iterative use of the Practical, Robust Implementation and Sustainability Model in public health and clinical settings Implement Sci Commun 2023 4 01 116 37726860 10.1186/s43058-023-00494-4 PMC10508024

[16] Dopp A R Parisi K E Munson S A Lyon A R A glossary of user-centered design strategies for implementation experts Transl Behav Med 2019 9 06 1057 1064 30535343 10.1093/tbm/iby119

[17] Trinkley K E Wright G Allen L A Sustained effect of clinical decision support for heart failure: a natural experiment using implementation science Appl Clin Inform 2023 14 05 822 832 37852249 10.1055/s-0043-1775566 PMC10584394

[18] Tirtanadi K Johnson K A Epler A J Chen J A Applications in human-centered design: shared-decision making for mental health treatment in primary care Patient Educ Couns 2025 136(108745(:108745 40139024 10.1016/j.pec.2025.108745 PMC12379738

[19] Dowell D Ragan K R Jones C M Baldwin G T Chou R CDC clinical practice guideline for prescribing opioids for pain—United States, 2022 MMWR Recomm Rep 2022 71 03 1 95 10.15585/mmwr.rr7103a1 PMC9639433 36327391

[20] Sommers S W Tolle H J Trinkley K E Clinical decision support to increase emergency department naloxone coprescribing: implementation report JMIR Med Inform 2024 12 e58276 39504560 10.2196/58276 PMC11560079

[21] Stevenson R Burnell D Fisher G The minimum viable product (MVP): theory and practice J Manage 2024 50 3202 3231

[22] Trinkley K E Kahn M G Bennett T D Integrating the practical robust implementation and sustainability model with best practices in clinical decision support design: implementation science approach J Med Internet Res 2020 22 10 e19676 33118943 10.2196/19676 PMC7661234

[23] Ray J Finn E B Tyrrell H User-Centered Framework for Implementation of Technology (UFIT): Development of an integrated framework for designing clinical decision support tools packaged with tailored implementation strategies J Med Internet Res 2024 26 e51952 38771622 10.2196/51952 PMC11150893

[24] Trinkley K E Maw A M Torres C H Huebschmann A G Glasgow R E Applying implementation science to advance electronic health record-driven learning health systems: case studies, challenges, and recommendations J Med Internet Res 2024 26 e55472 39374069 10.2196/55472 PMC11494259

[25] Smith J D Li D H Rafferty M R The Implementation Research Logic Model: a method for planning, executing, reporting, and synthesizing implementation projects Implement Sci 2020 15 01 84 32988389 10.1186/s13012-020-01041-8 PMC7523057

[26] Powell B J Waltz T J Chinman M J A refined compilation of implementation strategies: results from the Expert Recommendations for Implementing Change (ERIC) project Implement Sci 2015 10 01 21 25889199 10.1186/s13012-015-0209-1 PMC4328074

